# State-by-state estimates of avoidable trauma mortality with early and liberal versus delayed or restricted administration of tranexamic acid

**DOI:** 10.1186/s12873-022-00741-2

**Published:** 2022-12-03

**Authors:** Matthew J. Bivens, Christie L. Fritz, Ryan C. Burke, David W. Schoenfeld, Jennifer V. Pope

**Affiliations:** 1 Department of Emergency Medicine, Harvard Medical School, Beth Israel Deaconess Medical Center, MA Boston, USA; 2 Department of Emergency Medicine, Beth Israel Deaconess Medical Center, Boston, MA USA; 3 Department of Emergency Medicine, Geisel School of Medicine, Dartmouth-Hitchcock Medical Center, Lebanon, NH USA

**Keywords:** Trauma, EMS, Hemorrhage, Tranexamic acid (TXA)

## Abstract

**Objective:**

Early administration of tranexamic acid (TXA) has been shown to save lives in trauma patients, and some U.S. emergency medical systems (EMS) have begun providing this therapy prehospital. Treatment protocols vary from state to state: Some offer TXA broadly to major trauma patients, others reserve it for patients meeting vital sign criteria, and still others defer TXA entirely pending a hospital evaluation. The purpose of this study is to compare the avoidable mortality achievable under each of these strategies, and to report on the various approaches used by EMS.

**Methods:**

We used the National Center for Health Statistics Underlying Cause of Death data to identify a TXA-naïve population of trauma patients who died from 2007 to 2012 due to hemorrhage. We estimated the proportion of deaths where the patient was hypotensive or tachycardic using the National Trauma Data Bank. We used avoidable mortality risk ratios from the landmark CRASH 2 study to calculate lives saved had TXA been given within one hour of injury based on a clinician’s gestalt the patient was at risk for significant hemorrhage; had it been reserved only for hypotensive or tachycardic patients; or had it been given between hours one to three of injury, considered here as a surrogate for deferring the question to the receiving hospital.

**Results:**

Had TXA been given within 1 hour of injury, an average of 3409 deaths per year could have been averted nationally. Had TXA been given between one and three hours after injury, 2236 deaths per year could have been averted. Had TXA only been given to either tachycardic or hypotensive trauma patients, 1371 deaths per year could have been averted. Had TXA only been given to hypotensive trauma patients, 616 deaths per year could have been averted. Similar trends are seen at the individual state level. A review of EMS practices found 15 statewide protocols that allow EMS providers to administer TXA for trauma.

**Conclusion:**

Providing early TXA to persons at risk of significant hemorrhage has the potential to prevent many deaths from trauma, yet most states do not offer it in statewide prehospital treatment protocols.

**Supplementary Information:**

The online version contains supplementary material available at 10.1186/s12873-022-00741-2.

## Background

Trauma is the leading cause of death in the U.S. among people aged 1 to 44 [1]. It is the 4th leading cause of death overall, after heart disease, cancer and now COVID-19 [2].

Few dispute that time is critical in trauma care. Every state in the nation therefore has an emergency medical system (EMS) of prehospital providers, who respond rapidly to the scene, render initial care, and transport to definitive treatment. In potentially viable trauma cases transported by EMS, hemorrhage is a leading cause of mortality, accounting for an estimated 23-39% of deaths [3–8]. Interventions to treat hemorrhagic injuries are thus critical.

Tranexamic acid (TXA) is a generic anti-fibrinolytic agent that has been used for decades in elective surgeries. There are more than 129 randomized controlled trials (including 11 with pediatric patients) demonstrating it safely and reliably reduces bleeding [9]. In recent years it has been found to be life-saving for trauma patients. CRASH-2, the largest randomized trial in the history of trauma, showed that giving TXA to patients within 1 hour of injury reduced the risk of bleeding to death by 32%. When given within 3 hours of injury, there was no increased risk of pathological clotting identified [5]. Ker et al., using World Health Organization data, estimated that providing TXA to bleeding trauma patients worldwide within 1 hour of injury could save more than 128,000 lives a year, including nearly 4000 in the United States [8]. Critics of CRASH-2 have been skeptical of applying its results among a diverse international patient population to the United States, which has a robust trauma care system. An attempt to address that concern was the STAAMP trial, which randomized 903 U.S. trauma patients to prehospital TXA or placebo. Published in 2020, the trial was stopped early over funding shortfalls, was 20 times smaller than CRASH-2, and was underpowered to detect a difference in the primary outcome of overall mortality. However, while the study population was too small to achieve statistical significance, the trend toward survival among all comers treated with TXA was the same as in CRASH-2. Moreover, in a subgroup analysis of patients treated within 1 hour, STAAMP did find a statistically significant reduction in mortality of 40% (RR = 0.60, *p* < 0.002) [10]. This arguably validates the CRASH-2 findings for a U.S. trauma population: patients treated in either CRASH-2 or STAAMP within the first hour saw a significant survival benefit. In fact, the benefit was even stronger in the U.S. patients.

TXA’s benefit is highly time-sensitive. A meta-analysis of patients with severe bleeding from either traumatic injuries or post-partum hemorrhage found that immediate treatment with TXA improved chances of survival by about 70%, but that this survival benefit decreased by 10% for every 15 minutes of treatment delay — until there was no benefit at all seen after the 3-hour mark [11].

### Importance

Given that both trauma care itself, and TXA’s benefit in particular, are time-sensitive, TXA is a logical prehospital intervention. It is thus provided by civilian first responders from Israel to Germany to Canada, and recommended in major trauma by both the U.S. Department of Defense Committee on Tactical Combat Casualty Care and by the North Atlantic Treaty Organization (NATO) Blood Panel [12–14]. However, despite its safety, efficacy, and low cost (internationally, about $2.50 per 1 g dose intravenous) [15], prehospital TXA for trauma has not been broadly adopted in the United States [16, 17]. EMS systems in the United States are organized at the state or local level. Some prehospital treatment protocols allow for TXA in trauma cases that exhibit marked vital sign derangements such as hypotension or tachycardia. Others allow for TXA administration based on the prehospital clinician’s impression — informed by vital signs, exam, injury mechanism and overall presentation — that a patient is at high risk for significant hemorrhage. Still others do not include TXA for trauma and thus defer the question to the receiving hospitals.

### Goals of investigation

In this paper, we seek to illustrate the implications of various TXA treatment strategies. The specific aims were to calculate avoidable mortality estimates for three broad TXA administration scenarios and to describe current TXA protocols at the state level.

## Materials and methods

### Study design

This cross-sectional study used two national databases to estimate the potential avoidable mortality associated with different strategies for administering TXA to trauma patients. We used the CDC’s Underlying Cause of Death data, produced by the National Center for Health Statistics (NCHS), to estimate the number of blunt or penetrating trauma deaths in the United States overall, and individually for each state. We conducted a literature review to estimate the proportion of U.S. trauma deaths due to bleeding (Appendix 1). We then used estimates of 28-day mortality risk reduction drawn from the CRASH-2 study to calculate potential lives saved had providers administered TXA broadly in the first hour to major trauma patients; had providers reserved it only for patients who became hypotensive or tachycardic; or had TXA administration been deferred to one to three hours post injury.

Because the CDC dataset does not include vital signs for patients, we turned to the National Trauma Data Bank (NTDB), maintained by the American College of Surgeons (ACS), to capture more detailed information on trauma visits. We used the NTDB to calculate estimates for what percent of U.S. trauma patients in our TXA-naïve dataset presented with prehospital hypotension or tachycardia. We then applied this estimate to the Underlying Cause of Death data along with the CRASH 2 estimate of mortality risk reduction to calculate potential lives saved if providers administered TXA only to hypotensive or tachycardic patients.

We chose a specific six-year time period, 2007 to 2012, the most recent era when patients could safely be assumed to be “TXA naïve,” as this era pre-dated adoption of TXA use for trauma in either the prehospital or hospital settings.

### Underlying cause of death data

The Underlying Cause of Death Data was obtained via the CDC’s WONDER system. It is based on death certificate data from all 50 states and the District of Columbia. The data is available for U.S. residents only. Trauma deaths were compiled for 2007-2012, stratified by state. Query parameters were age > 15; place of death in a medical facility but not dead on arrival; and injury intent of unintentional, homicide, undetermined, or legal intervention/operations of war. Records were excluded if the injury mechanism was drowning, fire/flame, poisoning, or suffocation [18].

### Trauma deaths with associated hypotension or tachycardia

The NTDB is an aggregation of trauma registries across the United States, with participation from over 900 registered U.S. trauma centers [19]. The NTDB has more detailed patient-level data, including vitals from initial EMS presentation. Trauma deaths from the NTDB were included if the year of the trauma was 2007-2012. We included only deaths that occurred in the ED or the hospital. Death in the ED was defined as an ED disposition of “expired”. Death in the hospital was defined as the hospital disposition of “expired” and an ED disposition indicative of patient admission as an inpatient or observation. We only included traumas where the method of arrival to the ED was via EMS. Trauma deaths were excluded if the primary trauma type was not blunt or penetrating trauma, or if the age of the patient was less than 16. We defined hypotension as any EMS systolic blood pressure less than 90 mmHg. We defined tachycardia as any EMS heart rate greater than 110 beats per minute. Nearly half of records in the NTDB were missing EMS documentation of a blood pressure or heart rate. These EMS records were considered to be missing at random and were not included in the denominator.

### Trauma deaths due to bleeding

Not all blunt or penetrating trauma deaths are due to hemorrhage. We conducted a literature review to estimate what proportion of blunt and penetrating traumas likely died from bleeding. Estimates in the literature ranged from 23 to 39% (Appendix 1) [3–8]. We opted to estimate mortality from uncontrolled hemorrhaging on the lower end, so as to be more conservative in our estimates of potential benefit from TXA, and used 25%.

## Analysis

To estimate the total number of blunt or penetrating trauma deaths due to bleeding, we multiplied the trauma death estimates by 0.25. To estimate the number of these deaths potentially prevented had TXA been administered within one hour, we multiplied total deaths due to bleeding by 0.32, which was the estimated risk reduction from the CRASH-2 study when TXA was given within one hour of injury. To account for parameter uncertainty, we generated a range using the lower and upper bounds of the relative risk reduction confidence interval (0.18 and 0.43) [20].

To estimate the number of deaths averted had TXA been reserved only for patients with prehospital hypotension, or either prehospital tachycardia or hypotension, we multiplied the number of deaths due to bleeding by our estimated proportion of critical trauma patients in the NTDB who presented with those vital sign criteria. We then multiplied that smaller subset of trauma patients by the 0.32 risk reduction estimate to determine the number of lives saved if TXA had been given under those conditions. To account for parameter uncertainty, we generated a range using the lower and upper bounds of the relative risk reduction confidence interval (0.18 and 0.43) [20].

To estimate the number of deaths averted had TXA been administered during hours one through three, we multiplied total deaths due to bleeding by 0.21, which was the estimated risk reduction from the CRASH-2 study when TXA was given between hours one and three from time of injury. To account for parameter uncertainty, we generated a range using the lower and upper bounds of the relative risk reduction confidence interval (0.03 and 0.36) [20].

### State-level data

Using the state where the death occurred from the Underlying Cause of Death data, we generated the number of blunt or penetrating trauma deaths by state. We applied the same methodology to calculate state-level results. In addition, we conducted a review of state EMS protocols to determine the presence of TXA administration guidance. We classified each into three categories: statewide EMS protocol exists and includes TXA guidelines; statewide EMS protocol exists and does not include TXA guidelines; or no statewide protocol exists. The review was conducted either by downloading the official state treatment protocol, when available, or by contacting state governments to confirm absence of a statewide protocol. All protocols were reviewed in 2021 a final time prior to manuscript submission.

All analyses were conducted in SAS v9.4 and Microsoft Excel. This study was approved by the local Institutional Review Board.

## Results

**Fig. 1:**
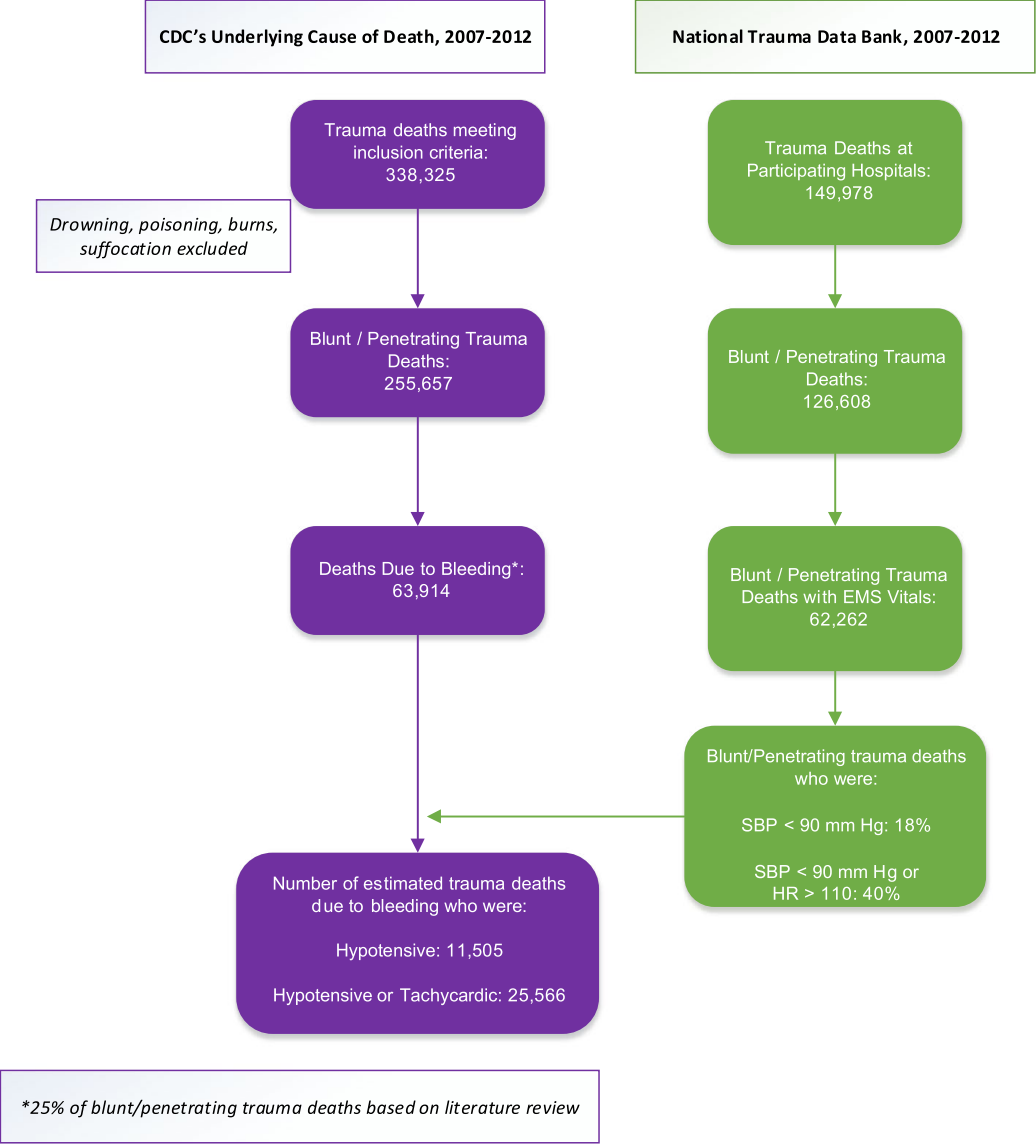
Flowchart of patients included in the study. CDC’s Underlying Cause of Death data (purple boxes) was used to identify adult deaths from blunt / penetrating trauma. A literature review (see appendix) was used to further estimate that 25% of those trauma deaths were due to bleeding. The National Trauma Data Bank (green boxes) is a smaller data set than the CDC’s but is more detailed and includes vital signs at presentation. It was used to estimate the percent of deaths from blunt or penetrating trauma that presented with hypotension and / or tachycardia

The number of trauma deaths identified from the Underlying Cause of Death data that met inclusion criteria were 338,325. After excluding 82,668 deaths not due to blunt or penetrating trauma, the number of deaths nationwide over the six-year study period was 255,657. The number of trauma deaths identified from the NTDB dataset that met inclusion criteria was 149,978. After excluding 16,126 deaths of persons under 16 years old and 7244 deaths not due to blunt or penetrating trauma, the number of deaths in the NTDB dataset was 126,608. See Fig. 1.

**Fig. 2:**
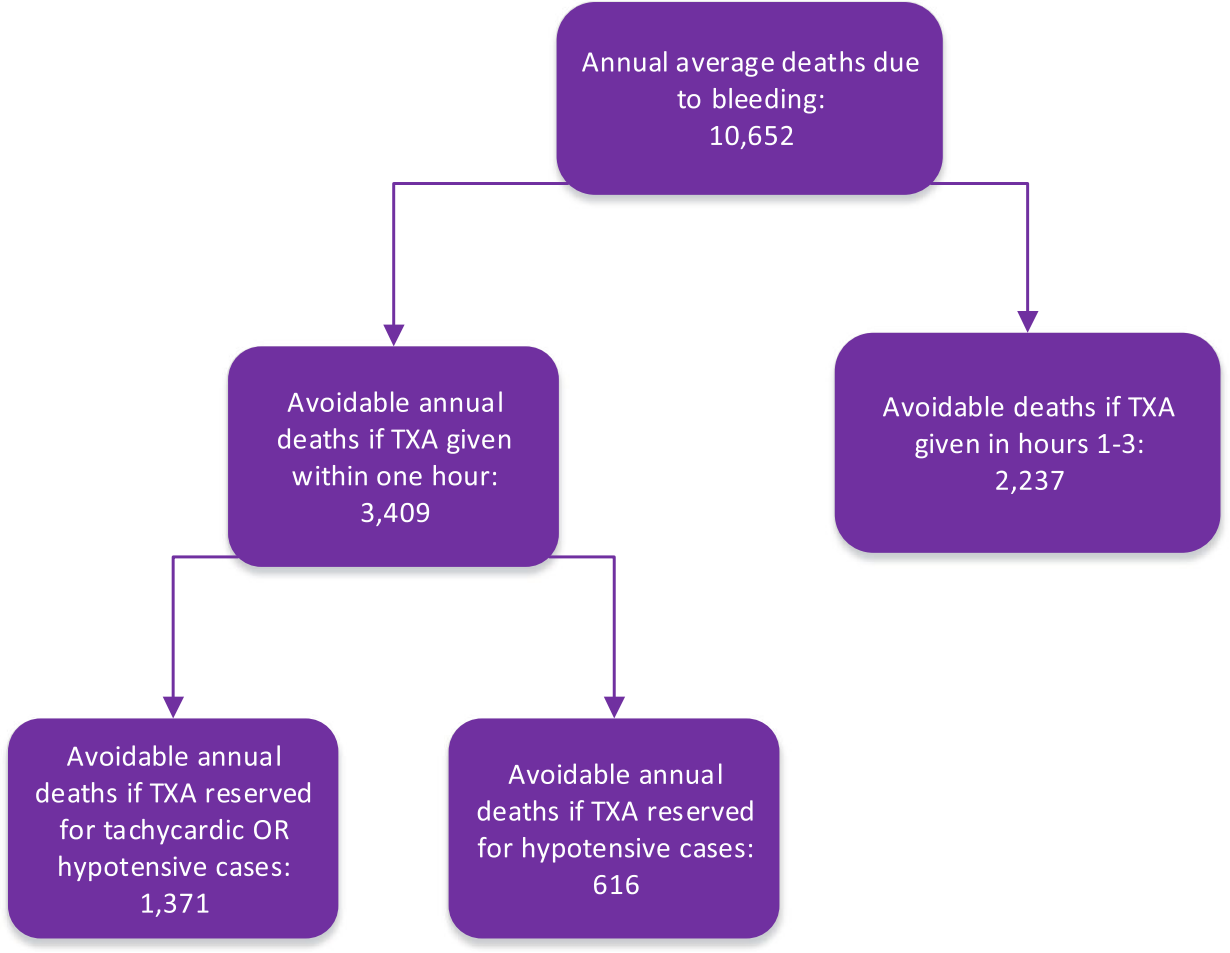
Avoidable mortality when TXA is given to severe traumas within one hour, which can be considered a surrogate for providing it in prehospital care; when it is only given between hours one and three, which can be considered a surrogate to deferring the decision to a hospital-based evaluation; or when it is provided only for patients with significant vital sign derangements

Yearly and average annual estimates of trauma deaths, and of potentially avoidable trauma deaths under various scenarios, are presented in Table 1 and also Fig. 2.

**Table 1:**
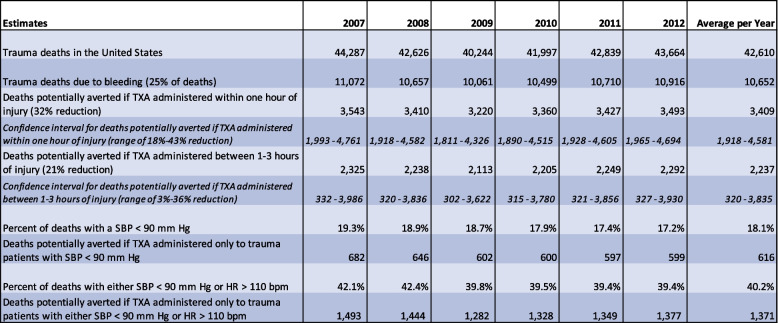
Study estimates for the number of trauma deaths avoided if TXA administered in the United States, 2007–2012

Data on all trauma deaths for 2007-2012, selected as a “TXA naïve” time period, and the subset of trauma deaths likely due to bleeding. It includes estimates of avoidable mortality if TXA had been administered within one hour of injury to all of these severe trauma patients; if it had been administered between one and three hours after injury; or if it had been reserved only for trauma patients found to be hypotensive, or only for trauma patients found hypotensive or tachycardic. The percent of deaths with hypotension and / or tachycardia were calculated from the NTDB. SBP = systolic blood pressure, HR = heart rate, bpm = beats per minute

The annual average of blunt or penetrating trauma deaths was 42,610. The annual average of such trauma deaths due to hemorrhage was 10,652.

If TXA had been administered within one hour of injury, then 3409 of trauma deaths due to bleeding (range: 1918-4581) could have been averted per year. If TXA had been administered between one and three hours of injury, then 2237 deaths (range: 320-3835) could have been averted per year. Stated another way: 1172 lives per year would be potentially lost by decisions to defer TXA until after the first hour.

The NTDB data indicated that 11,571 (18.1%) of visits would have been hypotensive in the prehospital setting and 26,644 (40.2%) of visits would have been tachycardic or hypotensive in the prehospital setting. If TXA had been administered in the first hour only for hypotensive or tachycardic patients, 1371 deaths (range: 767-1832) could have been averted per year. If TXA had been administered in the first hour only for hypotensive patients, 616 deaths (range: 347-829) could have been averted per year. Stated another way: 2793 lives per year would be potentially lost by decisions to reserve TXA only for hypotensive patients, and 2038 lives potentially lost by decisions to reserve TXA only for either hypotensive or tachycardic patients.

Avoidable annual mortality estimates by state are shown in Table 2.

**Table 2:**
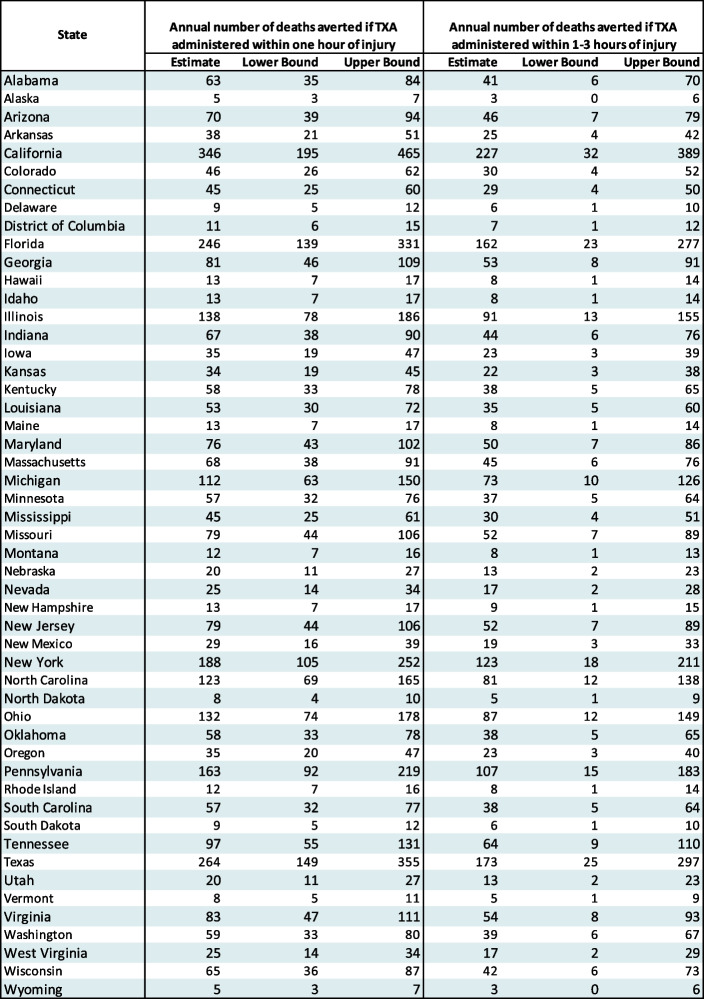
Annual number of trauma deaths avoided if TXA administered by state, 2002-2012

Annual avoidable mortality estimates broken down state by state if TXA is provided to major traumas either within the first hour, or between hours one and three, with the associated confidence intervals included

**Fig. 3:**
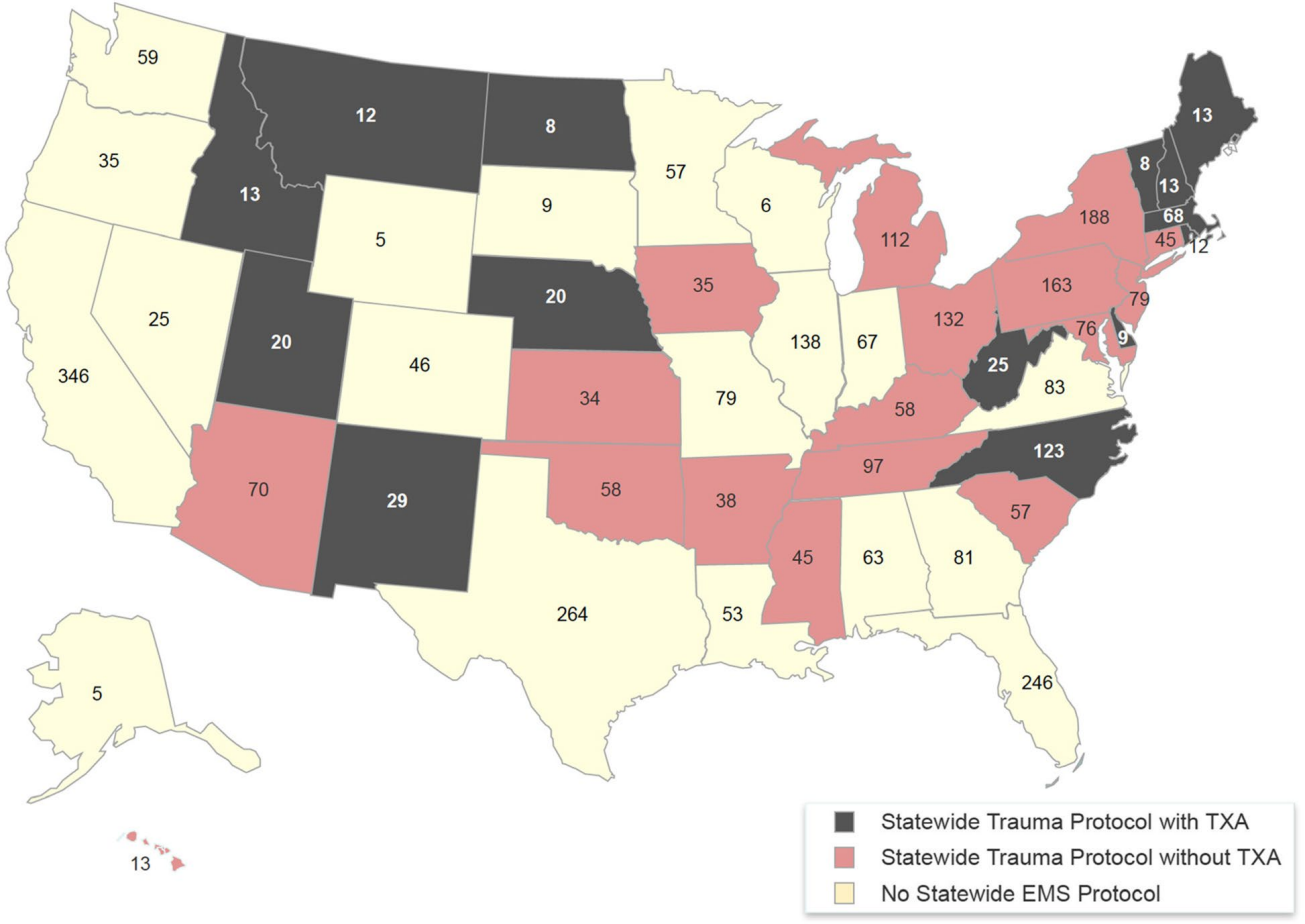
A visual guide to which states provide TXA in EMS trauma protocols. States pictured in black provide TXA in statewide trauma protocols. States pictured in red do not provide TXA in statewide trauma protocols. States pictured in yellow generally do not have prehospital treatment protocols at the state level (but may have local county or municipal treatment protocols). The number in each state is estimated avoidable mortality from providing TXA to major traumas within one hour of injury

A review of EMS treatment protocols showed 15 states have a statewide protocol that includes TXA, 18 states have a statewide protocol that does not include TXA, and another 17 states do not have state-level treatment protocols at all, instead leaving standing orders for EMS to be drafted at the county or municipal level. Fig. 3 maps protocol classification with the annual avoidable mortality estimate by state.

### Limitations

Our analysis of the national trauma datasets has several limitations. Perhaps the most significant is the overall approach of extrapolating findings from an international trial population to a national registry of contemporaneous U.S. trauma patients; and then, further extrapolating those findings forward in time to today, skipping over an era when trauma care evolved to include more use of tourniquets and blood products, less resuscitation with intravenous crystalloids, and other new approaches. This core analytical process of our paper thus has large inherent limitations. That said, it still does broadly illustrate the implications of prehospital trauma protocols to use liberally, use restrictively, or defer TXA entirely.

It is also encouraging for our approach that the STAAMP trial, 10 years after CRASH-2, found a statistically significant survival benefit among U.S. trauma patients treated with TXA within the first hour, and also among hypotensive traumatic shock patients. STAAMP was closed early due to funding and enrollment issues and is considered a negative trial because its primary outcome did not reach statistical significance. But the few trauma patients treated with TXA did survive in greater numbers than those who received placebo, and the improved survival rates were comparable to those in CRASH-2. Meanwhile, analysis of STAAMP subgroups — including the most important one for our purposes, trauma patients treated within one hour — actually saw statistically significant survival benefits with TXA. The STAAMP trial thus arguably validates CRASH-2’s core international findings in a U.S. trauma population, and further supports the use of CRASH-2’s estimates in our paper as we hazard broad estimates of the mortality implications of various TXA treatment protocols.

There are two other notable caveats inherent to our observational retrospective study. The first is that our study focuses only on trauma patients aged 16 and older. Trauma is a leading cause of pediatric deaths, but we did not attempt to calculate or estimate a survival benefit from use of TXA in pediatric trauma. This omission of many injured teenagers and children may underestimate potential mortality benefits from providing TXA to hemorrhaging trauma patients. The second caveat is that we cannot be certain no trauma patients in our dataset were treated with TXA. However, we chose a time period (2007-2012) largely prior to CRASH-2 (published in 2010), and national surveys at that time indicated little to no use of TXA for trauma prior to 2013 [21–23]. In fact, the absence of TXA use in trauma was so glaring, *The New York Times* in 2012 reported the fact as national news, writing, “[TXA’s] very inexpensiveness has slowed its entry into American emergency rooms … Because there is so little profit in it, the companies that make it do not champion it.”

We used CDC death certificate data to identify deaths occurring immediately after a blunt or penetrating trauma mechanism, and then used a literature review (see appendix) to estimate that 25% of those trauma patients died from hemorrhaging. This approach implicitly assumes that every death after a major trauma was traumatic, and ignores, for example, rare cases where a sudden-onset medical condition such as a myocardial infarction or hemorrhagic stroke precedes and actually causes a downstream trauma. The inclusion of these rare cases would slightly inflate our estimates, across all categories, of avoidable mortality with TXA administration.

We used the NTDB to estimate rates of critical trauma patients with hypotension or tachycardia in the field, but the NTDB is lacking EMS field documentation on nearly half of its patients. We assume these EMS charts are missing randomly, but there could be confounding reasons (for example, EMS could be unable to obtain vitals or information in critical cases; or unmotivated to complete timely charts in routine cases). We also cannot match vital signs obtained to causes of death. As discussed in Appendix 1, common causes of trauma mortality include central nervous system injuries, multi-organ failure, and hemorrhage. Only about 40% of trauma deaths reviewed here had hypotension or tachycardia in the prehospital environment, but it is logical that patients bleeding to death might make up a higher percentage of hypotensive traumas then, for example, those with CNS injuries. If so, as seems likely, then our approach may underestimate avoidable mortality from treating only hypotensive or tachycardic patients with TXA.

We assume that a patient transported by EMS to a hospital had at least some viable potential to survive. EMS systems as a rule do not transport patients with injuries obviously inconsistent with life; many per protocol do not transport traumatic cardiac arrest. Although we excluded patients classified as dead on arrival, some of the patients included here were possibly non-viable cases who EMS nevertheless transported and a trauma team nevertheless attempted to resuscitate. The inclusion of such patients would inflate our estimates, across all categories, of avoidable mortality with TXA administration.

We assume that if prehospital providers followed their clinical gestalt, then they would have been willing and able to provide TXA to every patient we identify as having died from hemorrhage. This further assumes that the prehospital team would include an advanced practitioner able to obtain intravenous access. This may overestimate the treatment benefit of a liberal protocol.

The CRASH-2 protocol called for an immediate TXA 1 g provided intravenously over 10 minutes, but also included a second 1 g infused over the next 8 hours. The STAAMP randomized controlled trial of prehospital TXA for select trauma found a statistically significant survival benefit in a subgroup that received additional in-hospital TXA [10]. It is unclear what the added benefit is of additional in-hospital TXA; if it is important to TXA’s survival benefit, but hospitals do not provide it, our approach may overestimate the benefits of prehospital TXA.

In addition to analyzing trauma datasets, we also surveyed national EMS practice patterns. Regarding our review of EMS trauma protocols, we only reviewed those at the state level, while 17 states have no state-level protocols whatsoever and defer treatment protocols to county or municipal levels. We did not investigate those 17 states more deeply and cannot say whether county or municipal protocols in those states include TXA for trauma.

## Discussion

This study used national datasets to identify a “TXA naïve” population of U.S. trauma patients, and then calculated estimates of avoidable mortality under various treatment strategies. The data suggest that 3409 deaths per year could have been prevented had EMS administered TXA early to these critical patients. This correlates well with a study from the same time period, published in *BMC Emergency Medicine* in 2012, that used World Health Organization data for the United States and calculated a range of 3497 to 3996 lives potentially saved with early TXA [8].

Other modeled scenarios that reserved TXA for hypotensive or tachycardic patients, or that delayed use until one to three hours from injury, resulted in fewer lives saved. Denying early TXA to trauma patients, who despite being critically ill nevertheless maintained systolic blood pressure readings above 90 mmHg, in our dataset led to 2739 potential lives lost annually. Deferring TXA to one to three hours post injury, which we considered here as a surrogate for deferring administration to a hospital, led to 1173 potential lives lost annually. These findings ripple out across all 50 states. They also track well with the results of the STAAMP trial, in which 903 prehospital trauma patients with hypotension or tachycardia were randomized to placebo or TXA. Mortality at 30 days in this U.S. trial trended similar to the far larger international CRASH-2 study (8.1% in patients receiving TXA vs 9.9% in patients receiving placebo), but the STAAMP trial was stopped early and did not achieve statistical significance. In a subgroup analysis of patients treated within one hour, however, the mortality difference in STAAMP was notably larger (4.6% vs 7.6%) and statistically significant (*P* < .002) [10]. An even more impressive mortality difference was found in another subgroup analysis, of trauma-associated shock, as defined by a systolic blood pressure < 70 mmHg (18.5% vs 35.5%, RR = 0.52, *p* < 0.003). TXA given rapidly in the field by U.S. paramedics thus might provide an even greater benefit than was seen in international settings. This could be related to the U.S. trauma care system’s excellent transportation infrastructure, which allows for bringing initial care, including TXA, rapidly to the patient in the field, and for equally rapid extrication to definitive care.

CRASH-2 eligibility was based on a treating clinician’s gestalt that the patient was “considered to be at risk of significant hemorrhage.” The study protocol provided vital sign parameters that might signal ongoing hemorrhage, but there were no required vital sign targets for enrollment [5]. Nevertheless, many proposals recommend reserving TXA for hypotensive and / or tachycardic trauma patients [12, 13, 22, 24–26]. It is true that TXA has been associated with powerful survival benefits in hypotensive trauma patients, as observed in the STAAMP trial [10] as well as in MATTERs [27] and MATTERs II [28], large observational studies of combat injury hospital admissions, and the PED-TRAX study [29], an observational study of pediatric traumas. While timely TXA clearly benefits a floridly exsanguinating patient, reserving it only for the most dramatic cases arguably misses most of the public health benefit. Roberts and Prieto-Merino, reviewing data from a U.K.-based trauma registry, note that TXA appears to reduce the risk of bleeding to death by about one-third, regardless of baseline risk. Timely administration would thus take a trauma patient with a 30% risk of bleeding to death to 20%; it would take a patient with a 3% risk of bleeding to death to 2%. They argue that because there are far more trauma patients with a baseline risk of death of 3% than of 30%, the potential for lives saved is largest among those who have injuries with lower (but real) potential for decompensation [30].

A feared complication of any medication to stanch bleeding is pathological clotting, yet there has been little pathological clotting identified with TXA use. A subgroup analysis of CRASH-2 patients who died from uncontrolled bleeding did find an increased risk of death when TXA was administered more than three hours after injury [20]. Disseminated intravascular coagulation (DIC) is a feared end-stage state of many prolonged life-threatening hemorrhages; the addition of a coagulation-supporting medication such as TXA to such cases may well cause harm. The HALT-IT trial of TXA use in gastrointestinal bleeding also found a 0.4% increase in venous thrombotic events, with no difference in mortality. The authors of HALT-IT noted that GI bleeding is often indolent for hours or days before it declares itself clinically, so this may again signal that TXA is best reserved for immediate use after acute bleeding [31]. Otherwise, across multiple large randomized trials powered to detect and motivated to look for pathological clotting, including in trauma patients [5, 10, 32–34], post-partum hemorrhages [35] and spontaneous intracranial hemorrhages [36], no increased risk of venous thrombosis, pulmonary embolism, or other coagulopathy has been seen. (See Table 3).

**Table 3:** Select Randomized Trials Studying Use of Tranexamic Acid

Title (Year published)	CRASH 2 (2010)	WOMAN (2017)	TICH-2 (2018)	CRASH 3 (2019)	STAAMP (2020)	HALT-IT (2020)	Prehospital TXA for TBI (2021)
Indication	Trauma patientswithin 8 hours of injury	Post-partum hemorrhage patients	Hemorrhagic stroke patientswithin 8 hours of symptom onset	TBI patients within 3 hours of injury	Hypotensive or tachycardic prehospital trauma patients	Upper or lower gastrointestinal bleeding patients	Severe TBI patients treated within 2 hours of injury
Dosing of TXA	1 g IV over 10 minutes, followed by 1 gm IV over 8 hours.	1 g IV over 10 minutes. If bleeding continued,or resumed in 24 hours, a second 1 gm might be administered.	1 g IV over 10 minutes, followed by 1 gm IV over 8 hours.	1 g IV over 10 minutes, followed by 1 gm IV over 8 hours.	1 g IV over 10 minutes, followed by either no further TXA, a second 1 g IV push, or a second 1 g IV over 8 hours.	1 g IV over 10 minutes, followed by 3 gms IV over 24 hours, "high-dose, 24-hour infusion".	Randomized 1:1:1 to placebo; 1 g IV over 10 minutes, followed by 1 gm IV over 8 hours; or 2 g IV over 10 minutes, followed by placebo infusion over 8 hours.
Number of patients	20,127	20,060	2,325	12,737	903	12,009	966
Outcomes	All-cause mortality reduced by 1.5% in patients treated (RR 0.91) at any point. Mortality due to bleeding reduced 2.4% (RR 0.68) if treated in first hour. Mortality due to bleeding reduced 1.3% (RR 0.79) if treated between hours one and two.	Mortality trend of 0.4% less in all patients treated with TXA was not statistically significant (p = 0.045). But all-cause mortality in patients treated within three hours of giving birth reduced by 0.5% and was statistically significant (p = 0.008, RR = 0.69).	No difference in mortality or functional outcome at 90 days. But statistically significant decrease in hematoma size, and early survival, favoring TXA.	Non-statistically significant trend toward reduced mortality with TXA. In large subgroup of 5,615 mild to moderate injuries, a statistically significant mortality benefit of 1.7% (RR 0.78).	Non-statistically significant trend toward reduced 30-day mortality with TXA. In pre-planned subgroup analyses, patients treated within one hour saw a statistically significant mortality benefit of 3% (RR = 0.60, p < 0.002); patients with severe shock (SBP < 70 mm Hg) saw a statistically significant mortality benefit of 17% (18.5% vs 35.5%; RR = 0.52, p < 0.003).	No difference in mortality at 5 days.	No difference in mortality at 28 days, disability at 6 months, or progression of hemorrhage.
Adverse Events	No increased risk of PE or DVT. In subset of patients who died from bleeding, increased risk of death among patients treated > 3 hours from time of injury.	No increased risk of PE or DVT.	No increased risk of PE or DVT.	No increased risk of PE or DVT.	No increased risk of PE or DVT.	0.4% increased risk of PE or DVT with TXA.	No increased risk of PE or DVT.

As an anti-fibrinolytic, TXA prevents the breakdown of fibrin clot. Viscoelastic tests such as thromboelastography (TEG) and rotational thromboelastometry (ROTEM) are often used to assess fibrinolysis status. Higher rates of fibrinolysis in trauma are associated with higher mortality. Some recommend obtaining viscoelastic testing in trauma patients prior to TXA administration in order to withhold TXA use among patients who exhibit hypofibrinolysis [22, 24, 25]. Others recommend a split-the-difference strategy of providing an empiric TXA 1 g in the field to high-risk trauma patients, followed by viscoelastic testing to determine whether to proceed in-hospital with an additional eight-hour TXA infusion [12, 13]. Despite the use of such strategies in some trauma centers, and important, ongoing work on viscoelastic testing to guide resuscitations, there is as yet no supporting randomized controlled trial data for these approaches. There have also not been any safety signals for TXA use across large trials when trauma patients were treated within three hours, which makes it even harder to justify delaying TXA treatment pending results of viscoelastic testing that, for this indication, remains experimental [37]. The science around the use of viscoelastic tests is new and evolving, as many of the lab values used to define hyper- or hypofibrinolysis remain unvalidated, and there is disagreement between published authorities on how to interpret or act upon the results [38, 39].

## Conclusion

This study illustrated the implications of various TXA treatment strategies. Based on the existing randomized controlled trial evidence, we already know that TXA given early saves lives and has an impressive safety. Guided by those trials it would already seem logical for prehospital providers to administer TXA to eligible trauma patients as soon as possible. Our study further illuminates the question by comparing the relative mortality costs of progressively more restrictive field treatment strategies — including the most restrictive of all, providing no TXA in the field and deferring the decision to hospital-based workups. We find more restrictive TXA strategies likely represent missed opportunities to save hundreds of lives every year, and have provided data on this down to the level of individual states to help guide EMS policy decision-making.

## Supplementary Information

**Additional file 1.**

## Data Availability

We analyzed two major national data sets for this study. The CDC’s Underlying Cause of Death data, produced by the National Center for Health Statistics (NCHS), is available in the CDC WONDER depository at https://wonder.cdc.gov/Deaths-by-Underlying-Cause.html The National Trauma Data Bank data are available from the American College of Surgeons, which provided the data for use under license for the current study. Applications to access the NTDB are available at https://www.facs.org/quality-programs/trauma/tqp/center-programs/ntdb/about
