# Supplementary material for: State-by-state estimates of avoidable trauma mortality with early and liberal versus delayed or restricted administration of tranexamic acid
Source: BMC Emerg Med. 2022 Dec 3;22:191. doi: 10.1186/s12873-022-00741-2 (PMC9719138; doi:10.1186/s12873-022-00741-2)
Supplement: Supplementary file 1 — Additional file 1. [file 12873_2022_741_MOESM1_ESM.docx]

**Appendix One: Literature Review on Trauma Death Rates Due to Bleeding**

The epidemiology of blunt and penetrating trauma has been studied nationally and internationally and for decades. Causes of death are usually categorized as due to central nervous system injuries (i.e., a traumatic brain injury); to hemorrhage; to multi-system organ failures; and to various less common causes, such as downstream sepsis or venous thromboembolic events.

Some studies report data on blunt and penetrating trauma as two separate entities. Most do not and report collective mortality for blunt and penetrating trauma.

Many studies cite the 1992 work of Sauaia et al., which looked in detail at 289 lethal injuries in their Denver-based trauma system and found that, in blunt and penetrating trauma deaths, 39% had died of bleeding.^3^ The authors noted they were reprising a landmark 1977 San Francisco study, and they reported they had found similar injury mechanisms, demographics and causes of death: “CNS injuries (San Francisco = 50% vs. Denver = 42%) and exsanguination (San Francisco = 31% vs. Denver = 39%) were the primary causes of death in both communities.”

These findings that CNS injuries such as traumatic brain injuries are a leading cause of death, followed closely by hemorrhage, are echoed in other literature. A rough range of 25-35% of blunt and penetrating trauma deaths being due to bleeding has been put forward across multiple more recent studies.

The CRASH-2 study, a randomized controlled trial with more than 20,000 patients worldwide, calculated

a 35% rate of bleeding deaths in trauma.^5^

Dutton et al, looking at more than 68,000 admissions to the Baltimore-based Shock Trauma Center over a 12-year period (1997-2008), found that acute hemorrhage accounted for 30% of all trauma deaths.^4^

Oyeniyi et al reviewed 1,029 blunt and penetrating trauma deaths in a Houston-based trauma system over two time periods, from 2005-2006 and 2012-2013, and found deaths from hemorrhaging fell from 36% to 25% over time. In part, they hypothesize, this may have been due to an increased focus on hemorrhage control interventions.^6^

More recently, Callcut et al reported on prospective data collected across 18 U.S. trauma centers on 1,536 blunt and penetrating trauma cases from 2015 to 2017. They found, again, traumatic brain injury to be the leading cause of death at 45%, followed by exsanguination at 23%.^7^

Some investigators have further parsed the data to come up with rates of exsanguination in blunt trauma vs. penetrating trauma as separate categories. Ker et al. performed a 2012 literature review that identified 14 studies with data on 24,831 trauma deaths, including in-hospital trauma death data. They then further narrowed that to 5 studies involving 9,684 deaths that presented data on the proportion of blunt trauma deaths due to hemorrhage, and four studies with data on proportion of penetrating trauma deaths due to hemorrhage.^8^ The pooled proportions suggested 18% of blunt trauma deaths were due to hemorrhage and 55% of penetrating trauma due to hemorrhage. These are similar to the findings of Dutton et al., which found 18.5% of blunt trauma deaths in Baltimore due to hemorrhage and 46.7% of penetrating trauma due to hemorrhage; and to Callcut et al., who in their data collected across 18 trauma centers found 12.5% of blunt trauma deaths due to hemorrhage and 51.7% of penetrating trauma due to hemorrhage.

Of note, neither the CRASH 2 study nor most state-level prehospital treatment protocols draw a distinction between blunt and penetrating trauma, and instead consider both as having potential for serious bleeding injuries. Here we thus consider accidental blunt and penetrating trauma as a single clinical entity, and assumed that 25% of mortality involved uncontrolled hemorrhaging.
